# Population-based study of the reproductive risk factors for transvaginal ultrasound diagnosed uterine fibroids in Nigerian women

**DOI:** 10.1038/s41598-023-44703-5

**Published:** 2023-11-02

**Authors:** Clement A. Adebamowo, The ACCME Research Group as part of the H3Africa Consortium, Sally N. Adebamowo, Richard Offiong, Olayinka Olaniyan, Kayode Obende, Amos Adebayo, Sanni Ologun, Bunmi Alabi, Peter Achara, Juliet Iyosaba Erhunmwonsere, Yinka Owoade, Tolu Gbolahan, Sally N. Adebamowo

**Affiliations:** 1 Department of Epidemiology and Public Health, and the Greenebaum Comprehensive Cancer Center, University of Maryland School of Medicine, 660 West Redwood Street, Baltimore, MD 21201 USA; 2 Center for Bioethics and Research, Ibadan, Nigeria; 3 Institute of Human Virology Nigeria, Abuja, Nigeria; 4 University of Abuja Teaching Hospital, Gwagwalada, Abuja, Nigeria; 5 National Hospital Abuja, Abuja, Nigeria; 6 Garki Hospital Abuja, Abuja, Nigeria; 7 Asokoro District Hospital, Abuja, Nigeria; 8 Kubwa General Hospital, Abuja, Nigeria; 9 Wuse General Hospital, Abuja, Nigeria; 10 Federal Medical Center, Keffi, Nigeria

**Keywords:** Reproductive signs and symptoms, Urogenital diseases, Risk factors

## Abstract

There has been no previous systematic, epidemiological study of the reproductive risk factors for uterine fibroids (UF) in African populations despite African women having the highest burden of UF in the world. Improved knowledge of the associations between UF and reproductive factors would contribute to better understanding of the etiology of UF and may suggest novel opportunities for prevention and therapeutic interventions. We used nurse administered questionnaires to survey the demographic and reproductive risk factors of UF among 484 women who are members of the African Collaborative Center for Microbiome and Genomics Research (ACCME) Study Cohort in central Nigeria, and who had transvaginal ultrasound diagnosis (TVUS). We used logistic regression models to the evaluate associations between reproductive risk factors and UF, adjusted for significant covariates. In our multivariable logistic regression models, we found inverse associations with number of children (OR = 0.83, 95%CI = 0.74–0.93, p-value = 0.002), parity (OR = 0.41, 95%CI = 0.24–0.73, p-value = 0.002), history of any type of abortion (OR = 0.53, 95%CI = 0.35–0.82, p-value = 0.004), duration of use of Depot Medroxyprogesterone Acetate (DMPA) (p-value for trend = 0.02), menopausal status (OR = 0.48, 95%CI = 0.27–0.84, p-value = 0.01), and a non-linear positive association with age (OR = 1.04, 95%CI = 1.01–1.07, p-value = 0.003). Other reproductive risk factors that have been reported in other populations (age at menarche and menopause, and oral contraceptives) were not associated with UF in this study. Our study confirms some of the reproductive risk factors for UF that have been found in other populations and shows that some of them are stronger in the Nigerian population. The associations we found with DMPA suggest opportunities for further research to understand the mechanisms of action of progesterone and its analogues in the etiology of UF, their potential use for prevention and treatment of UF.

## Introduction

African women have some of the highest prevalence of uterine fibroids (UF) in the world where it is associated with significant morbidity, economic and health systems costs, and occasional mortality^1–6^. The reported incidence and prevalence of UF varies significantly globally, by study design, method of diagnosis, age distribution, racial and ethnic composition of study participants^1,7^. Among women in High-Income Countries (HIC), the cumulative incidence of UF by the age of 50 years ranges from 70 to 80%^1,8^. We previously showed that previous studies of UF in Africa were facilities-based, lacked adequate power, and had poor study design^2^. In our population based study of Nigerian women who were participating in a prospective cohort study, we diagnosed UF using transvaginal ultrasound, and found a prevalence of 45.1%^3^.

Several studies in other populations suggest associations between reproductive factors and UF^9,10^. High parity, early age at menarche and at first birth have been inversely associated with risk of UF, while age at last full term birth is positively associated with UF^7,11–14^. Though both estradiol and progesterone are critical for development of UF, the association with oral contraceptives remains unsettled. Some papers report inverse associations while others found positive or no associations^1,7,9,15^. Hormone replacement therapy in postmenopausal women was associated with increased risk of UF in some studies^7^. Studies have also shown interactions between reproductive risk factors and race in admixed societies like the US^7,16^. While the growth rate of UF tends to decline in white women as they approach menopause, similar decline has not been observed in black women^16^.

In this paper, we report our adequately powered, systematic, population-based study of the associations between reproductive risk factors and transvaginal ultrasound diagnosed UF in Nigerian women.

## Methods

The ACCME cohort study profile and the characteristics of the study participants have been previously described in detail^17^. We enrolled 11,700 women who were at least 18 years old at baseline, HIV negative, and had a history of penetrative vaginal intercourse but no previous history of cervical abnormalities, cervical cancer, or hysterectomy into the ACCME cohort from 2014 to 2016. Trained research nurses administered validated questionnaires to collect demographic, lifestyle, obstetric and gynecologic history, medical history, sexual behavior and practices, diet, and physical activities.

In 2020, for this study, we developed and internally validated a supplemental questionnaire among 20 ACCME participants, who were not included in the rest of the UF study. The questions were:

- Has a doctor ever told you that you have uterine fibroid and you have not received any treatment for it?
- Has a doctor ever told you that you do not have uterine fibroid?
- Have you ever been told that you have a uterine fibroid on abdominal ultrasound scan and have not received treatment for it?
- Have you ever been told that you do not have uterine fibroids on abdominal ultrasound scan?
- Do you have any symptom—excessive bleeding during menstrual periods, severe pelvic/abdominal pain, inability to conceive, abdominal swelling—that have been attributed to uterine fibroids?

Using random numbers generated with Stata software, we identified and invited 486 women from the ACCME cohort to participate in this study. All those invited responded and consented. We retrieved the participants’ epidemiological data from the ACCME Cohort baseline dataset. The variables we selected included age in years, years of education completed (no formal education, 1–6 years of schooling, 7–12 years of schooling, any schooling after 12 years including university education, higher diplomas, etc.), occupation, religion, and marital status (married, unmarried). To compute socio^®^-economic status (SES), we calculated the ‘wealth index’ using the following variables: house ownership and type of house owned (e.g., home, apartment, house, or duplex); source of drinking water (e.g., from outside, well, borehole, piped or bottled); type of cooking fuel; use of separate room for cooking; type of toilet; and ownership of household goods including car and refrigerator. We used principal component analysis (PCA) with varimax rotation to compute factor scores based on the sum of responses to these variables weighted by their factor loading. We used the first component in the PCA that explained 35% of the variation in the data to generate a wealth index (39). The wealth index variable was used to classify participants into low SES (lowest 50% of the score distribution), middle (middle 30%) and high (highest 20%) socioeconomic classes.

We also retrieved data on reproductive risk factors including age at menarche, age at sexual debut, parity, history of spontaneous or induced abortion, history of ectopic pregnancies, age at menopause, menopausal status (premenopausal vs. postmenopausal—individuals who reported that they were not sure of their menopausal status were classified as post-menopausal in multivariable analyses), stillbirths, history of regular contraceptive use (yes vs. no), age at commencement of regular contraceptive use, and duration of use of either oral contraceptives, Depot Medroxyprogesterone Acetate (DMPA), or contraceptive implants.

Participants were offered transvaginal ultrasound scan (TVUS) performed by clinical radiologists whose fellowship was certified by the West African College of Surgeons. UF identification was based on the Muram criteria and included all relatively spherical, echogenically distinct masses in the myometrium that were more than 0.5 cm in diameter^18^. Participants with UF on TVUS were referred to the gynecologic clinic for standard of care treatment.

We used the Table 1 command in Stata 18^®^ to generate our table of descriptive statistics showing distribution of variables among cases and controls, and p-values for chi-square tests of categorical variables and t-tests of continuous variables comparing women with UF and those without. To construct our regression models, we identified variables with *p-values* < 0.20 in age-adjusted analyses and included them in our multivariable logistic models. We used restricted cubic splines to explore continuous variables such as age at menarche, age at sexual debut, and age at menopause for potential non-linear relationships with risk of UF. In the multivariable models, we used Wald test to identify covariates with significant associations with risk of UF at *p-value* < 0.05. We analyzed all data using Stata 18^®^ (College Station, Texas 77845 USA).

**Table 1:** Baseline characteristics of women with TVUS diagnosed UF and those without TVUS diagnosed UF, Nigeria, 2021.

	TVUS diagnosis of uterine fibroid			
	Negative	Positive	Total	p-value
	(N = 266)	(N = 218)	(N = 484)	
	Mean (SD) N (%)	Mean (SD) N (%)	Mean (SD) N (%)	
Age (years)	37.1 (9.35)	36.9 (9.03)	37.0 (9.20)	0.88
Age groups				
18–29	64 (24.1%)	50 (23.1%)	114 (23.7%)	0.42
30–39	90 (33.8%)	85 (39.4%)	175 (36.3%)	
40–49	89 (33.5%)	59 (27.3%)	148 (30.7%)	
50 and above	23 (8.6%)	22 (10.2%)	45 (9.3%)	
Education				
No formal schooling	25 (9.4%)	22 (10.1%)	47 (9.7%)	0.04
1–6 years of schooling	46 (17.3%)	20 (9.2%)	66 (13.6%)	
7–12 years of schooling	73 (27.4%)	54 (24.8%)	127 (26.2%)	
University	122 (45.9%)	122 (56.0%)	244 (50.4%)	
Occupation				
Unemployed	42 (15.8%)	40 (18.3%)	82 (16.9%)	0.46
Employed	224 (84.2%)	178 (81.7%)	402 (83.1%)	
Religion				
Islam	55 (20.7%)	51 (23.4%)	106 (21.9%)	0.47
Christian	211 (79.3%)	167 (76.6%)	378 (78.1%)	
Marital status				
Unmarried	39 (14.7%)	47 (21.6%)	86 (17.8%)	0.048
Married	227 (85.3%)	171 (78.4%)	398 (82.2%)	
Socioeconomic status				
Low SES	126 (47.4%)	108 (49.5%)	234 (48.3%)	0.85
Middle SES	88 (33.1%)	67 (30.7%)	155 (32.0%)	
High SES	52 (19.5%)	43 (19.7%)	95 (19.6%)	

### Patient and public involvement

Patients and the public were not involved in the design and analysis of this project. The study is population based and thus did not enroll individuals who presented as patients in a medical institution. The findings on TVUS were shared with participants who were counselled on accessing the standard of care for UF in Nigeria. The idea and design of the study was based on clinical experience with patients, but these patients were not involved in the recruitment or conduct of this study.

### Ethics approval

The study protocol was reviewed and approved by the National Health Research Ethics Committee of Nigeria (NHREC Approval Number NHREC/01/01/2007-29/11/2016) and an IRB authorization agreement with the University of Maryland School of Medicine. All research methods were performed in accordance with the relevant clinical and diagnostic guidelines in Nigeria. ACCME study participants receive the project newsletter quarterly, and the results of this study are shared with them through this mechanism. The ACCME Project has an active website and social media that is used to engage study participants and disseminate the results of this study. This website is also used to grant controlled access to data used in this study.

## Results

Of the 486 women invited, there was complete data for analysis in 484 women. The mean (SD) age of all participants in this study was 37.0 (9.20) years, and this was not significantly different comparing women with UF (mean = 36.9, SD = 9.03) with those without UF (mean = 37.1, SD = 9.35) (*p-value* = 0.88). Women with UF were more likely to have received more formal schooling (*p-value* = 0.04) and have single marital status (*p-value* = 0.048) compared to women without UF (Table 1).

### Reproductive risk factors and UF (Table 2)

**Table 2:** Reproductive risk factors for TVUS diagnosed UF, Nigeria, 2021.

	TVUS diagnosis of uterine fibroid			
	Negative	Positive	Total	p-value
	(N = 266)	(N = 218)	(N = 484)	
	Mean (SD) N (%)	Mean (SD) N (%)	Mean (SD) N (%)	
Age at menarche (years)	14.3 (1.97)	14.3 (1.93)	14.3 (1.95)	0.97
Age at menarche categories				
8–14	148 (55.8%)	124 (57.4%)	272 (56.5%)	0.91
15–19	111 (41.9%)	88 (40.7%)	199 (41.4%)	
20–24	6 (2.3%)	4 (1.9%)	10 (2.1%)	
Age at sexual debut	19.8 (3.87)	20.1 (3.95)	19.9 (3.90)	0.38
Age at sexual debut categories				
Less than 15	10 (3.8%)	11 (5.1%)	21 (4.4%)	0.58
15–19	124 (46.8%)	90 (42.1%)	214 (44.7%)	
20–24	88 (33.2%)	81 (37.9%)	169 (35.3%)	
25 and above	43 (16.2%)	32 (15.0%)	75 (15.7%)	
Number of children	3.12 (1.97)	2.62 (1.99)	2.90 (1.99)	0.006
Parity status				
Nulliparous	28 (10.5%)	43 (19.7%)	71 (14.7%)	0.004
Parous	238 (89.5%)	175 (80.3%)	413 (85.3%)	
Abortion				
No	176 (66.2%)	165 (75.7%)	341 (70.5%)	0.02
Yes	90 (33.8%)	53 (24.3%)	143 (29.5%)	
Ectopic pregnancy				
No	265 (99.6%)	218 (100.0%)	483 (99.8%)	0.37
Yes	1 (0.4%)	0 (0.0%)	1 (0.2%)	
Age at menopause	44.9 (5.44)	45.8 (5.38)	45.2 (5.40)	0.49
Menopause				
No	199 (75.1%)	176 (81.5%)	375 (78.0%)	0.09
Yes	66 (24.9%)	40 (18.5%)	106 (22.0%)	
Stillbirth				
No	253 (95.1%)	212 (97.2%)	465 (96.1%)	0.23
Yes	13 (4.9%)	6 (2.8%)	19 (3.9%)	
Regular use of contraceptive methods				
No	143 (53.8%)	141 (64.7%)	284 (58.7%)	0.02
Yes	123 (46.2%)	77 (35.3%)	200 (41.3%)	
Age at regular use of contraceptive methods	28.3 (6.13)	29.0 (5.98)	28.6 (6.07)	0.42
Duration of oral contraceptive use				
Never used	222 (83.5%)	189 (86.7%)	411 (84.9%)	0.21
< 1 year	21 (7.9%)	19 (8.7%)	40 (8.3%)	
=/> 1 year	23 (8.6%)	10 (4.6%)	33 (6.8%)	
Duration of DMPA use				
Never used	212 (79.7%)	200 (91.7%)	412 (85.1%)	< 0.001
< 1 year	20 (7.5%)	5 (2.3%)	25 (5.2%)	
=/> 1 year	34 (12.8%)	13 (6.0%)	47 (9.7%)	
Duration of contraceptive implant use				
Never used	243 (91.4%)	203 (93.1%)	446 (92.1%)	0.26
< 1 year	10 (3.8%)	3 (1.4%)	13 (2.7%)	
=/> 1 year	13 (4.9%)	12 (5.5%)	25 (5.2%)	

Women without UF had more children (mean = 3.12, SD = 1.97 compared to mean = 2.62, SD = 1.99; *p-value* = 0.006), were less likely to be nulliparous (*p-value* = 0.004), more likely to have had any type of abortion (*p-value* = 0.02), more likely to have regularly used any form of contraception (*p-value* = 0.02), and were more likely to have used DMPA for at least a year or more (*p-value* < 0.001).

There were no significant associations between age at menarche, age at sexual debut, age at regular use of any form of contraception, history of ectopic pregnancy, age at menopause, menopausal status, stillbirths, duration of use of oral contraceptives, and duration of use of contraceptive implants, and TVUS diagnosed UF.

### Multivariable analyses (Table 3)

**Table 3:** Multivariable analyses of reproductive risk factors for TVUS diagnosed UF, Nigeria, 2021.

	Age-adjusted model		Multivariable model	
	OR (95% CI)	P-value	OR (95% CI)	P-value
Age	1.00 (0.98–1.02)	0.88	1.04 (1.01–1.07)	0.003
Number of children^a^	0.85 (0.76–0.94)	0.002	0.83 (0.74–0.93)	0.002
Parity status^a^				
Nulliparous (ref)				
Parous	0.45 (0.27–0.77)	0.004	0.41 (0.24–0.73)	0.002
Abortion				
No (ref)				
Yes	0.64 (0.42–0.95)	0.03	0.53 (0.35–0.82)	0.004
Duration of DMPA use		0.001^**◊**^		0.02^**◊**^
Never used (ref)				
< 1 year	0.26 (0.10–0.72)	0.009	0.28 (0.10–0.78)	0.02
=/> 1 year	0.40 (0.20–0.79)	0.008	0.53 (0.26–1.07)	0.08
Menopause				
No (ref)				
Yes	0.61 (0.37–1.02)	0.06	0.48 (0.27–0.84)	0.01
Socio-economic status		0.87^**◊**^		
Low (ref)				
Middle	0.91 (0.60–1.37)	0.65		
Upper	0.99 (0.60–1.61)	0.96		
Education^b^		0.06^**◊**^		
No formal schooling (ref)				
1–6 years of schooling	0.50 (0.23–1.08)	0.08		
7–12 years of schooling	0.81 (0.41–1.58)	0.53		
University	1.14 (0.61–2.14)	0.68		
Marital status^b^				
Unmarried (ref)				
Married	0.62 (0.38–0.99)	0.045		
Regular contraceptive use^b^				
No (ref)				
Yes	0.63 (0.43–0.91)	0.01		

^a^These variables were replaced for each other in the multivariable models and both were not simultaneous included in same models.

^b^Variables did not achieve significance in multivariable models and were dropped.

^◊^p-value for trend.

In age-adjusted logistic regression models, parity, menopausal status, history of any type of abortion, history of regular use of any form of contraception, and duration of use of DMPA were significantly associated with history of TVUS diagnosed UF. However, only age, history of any type of abortion, duration of use of DMPA, and menopausal status were significantly associated with UF in multivariable logistic regression models. Marital status and history of regular use of contraceptives did not retain the associations observed in age-adjusted models. Education was marginally associated with UF in age-adjusted models but not in multivariable models.

While socio-economic status (SES) was not significantly associated with UF in age-adjusted and multivariable models, we retained it in the multivariable models because of its potential to account for unmeasured confounding. We conducted likelihood ratio test of the model with SES and that without SES and there was no significant difference (*p-value* = 0.73), so we removed SES from our final model.

**Figure 1:**
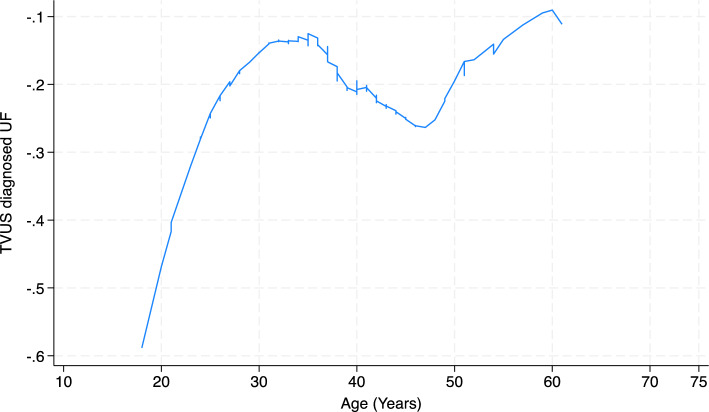
Relationship between age and risk of TVUS diagnosed UF, Nigeria, 2021.

Age became strongly associated with UF in multivariable logistic regression models whereas it was not associated when it was regressed against UF thus suggesting that the association may be nonlinear, or the association with age became more obvious after adjustment for confounders. To check the linearity of the relationship between age and UF, we estimated a locally weighted scatterplot smoothing (LOWESS) curve of the relationship between age and UF in the logistic regression model^19^. Fig. 1 shows the graphical relationship suggesting increasing association with UF till age 35 followed by a shallow decline and a subsequent rise in risk of UF with increasing age.

## Discussion

In this first systematic epidemiological study of the associations between reproductive risk factors and TVUS diagnosed UF in a population-based cohort of Nigerian women, we found associations with parity, abortion, regular use of DMPA for contraception, menopausal status, and a non-linear association with age. Other reproductive risk factors that have been reported in other populations (age at menarche and menopause, and oral contraceptives) were not associated with UF in this study^1,2,7,13^. Previous studies of reproductive risk factors for UF in African populations showed associations with age and parity^20–22^. However, none of the previous studies in Africa were population-based and they did not use validated tools for diagnosis of UF. They were also often poorly designed and inadequately powered^2^.

Several studies have shown an association between parity and UF but this is the first study to show similar and stronger association in African women compared to women with non-African ancestries^7,23^. In this study, we found 17% reduction in risk of UF for each child and 59% reduction in risk of UF comparing parous with nulliparous women^7^. These risk reductions are substantially higher than the 20% to 50% risk reductions previously reported from studies conducted in other populations^7^. There are multiple potential mechanisms whereby parity may influence the development and growth of UF. These include direct effect of pregnancy associated hormones on the myometrium and the extracellular matrix that constitutes UF, and non-hormonal mechanisms^7,10,11,15,24^. Fibroids may also involute during the remodeling of the uterus, post-partum^25^.

The history of any type of abortion was associated with reduced risk of UF by about 17% in this study. This effect may be independent of the effect of parity. Previous studies have reported conflicting associations between abortions and UF^7,24,26^. While some have suggested that a causal relationship between spontaneous abortions and UF is questionable, there is little doubt that ascertaining causality or reverse causality in this association would be challenging, even for the most robustly designed and implemented study^27^. This finding may be a reflection of the association between pregnancies and UF in general and not specific effect of abortions on risk of UF.

We found a marginal association between regular use of any type of contraception and UF, but this did not persist in multivariable models. This may be because more granular data on type and duration of use of different contraceptives than what is available in our study is required to adequately capture this exposure. There was no association with use of oral contraceptives. Most of the previous studies of oral contraceptives and UF have shown either decreased or null associations^7,28,29^. There are serious methodological challenges with research on use of oral contraceptives in sub-Saharan Africa where uptake is low, there are variations in the compositions of oral contraceptives sold, and adherence with regular intakes is uncertain^30,31^. We found significant association with use of DMPA and this declined with increasing duration of use in multivariable models. This is similar to findings in other populations^7,15,32–34^. DMPA may be associated with reduced risk of UF due to estradiol mediated reduction in progesterone receptor expression or differences in the mechanisms of action of exogenous compared with endogenous progesterone^34^.

The risk of UF in our study rose rapidly from early adulthood to about 35 years of age after which it declined slightly, then it resumed a gentler increase in risk. This finding is consistent with the pattern of change in reproductive risk factors with aging and the findings from other studies, including cohort studies that reported peak age incidence of 40–44 years^35^. The earlier peak age in this study may be a reflection of the population distribution of the underlying study population and is not an age incidence, therefore, it should not be compared with results from cohort studies. Nevertheless, recent global studies of UF suggest that the associations of UF with age peaked at 35 to 44 years, then declined with advancing age^36,37^. There was a 53% reduction in the risk of UF at menopause in this study. The natural regression of fibroids at menopause has been established in several studies and this has been attributed to the changes in estrogen and progesterone levels that occur at menopause^7,38^.

Despite our large sample size, population-based enrollment of study participants, and use of TVUS, our study has some limitations. We used data from questionnaires that were administered before we enrolled participants for the UF study. While this may reduce recall bias, the life course of UF is dynamic and may have changed in individual participants between enrollment into the ACCME study and TVUS. Large, prospective cohort studies in African populations are required to adequately account for secular trends in life-course of UF and their relationships with risk factors. We did not have data on all the reproductive risk factors for UF. Future studies focused on UF may seek to elicit more detailed responses but at the risk of over-burdening participants and reducing the quality of responses.

## Conclusions

In this study, we confirmed many of the associations between UF and reproductive risk factors that have been described in other populations for the first time in a population-based, study of TVUS diagnosed UF in an African population. This is important because African women bear the highest burden of UF in the world. Studies like ours improve understanding of the risk of UF which may lead to identification of biomarkers and therapeutic targets. They also create opportunities for early detection and preventive interventions.

## Data Availability

The data will be made available upon request from the corresponding author.
